# Aeromedical retrieval services characteristics globally: a scoping review

**DOI:** 10.1186/s13049-022-01053-x

**Published:** 2022-12-12

**Authors:** Kuda Muyambi, Fergus Gardiner, Stephen Sollid, Per Kristian Hyldmo, Engida Yisma, Breeanna Spring, Per Bredmose, Martin Jones, Sandra Walsh, Zoe Schofield, Marianne Gillam

**Affiliations:** 1 Department of Rural Health, University of South Australia, Adelaide, Australia; 2 IIMPACT in Health, University of South Australia, Adelaide, South Australia Australia; 3 Royal Flying Doctor Service, Canberra, Australia; 4 Australian National University, Canberra, Australia; 5 University of Stavanger, Stavanger, Norway; 6 Division of Prehospital Services, Air Ambulance Department, Oslo University Hospital, Oslo, Norway; 7 Division of Prehospital Care, Sørlandet Hospital, Sørlandet, Norway; 8 Charles Darwin University, Casuarina, Australia; 9 Norwegian Air Ambulance Foundation, Oslo, Norway

**Keywords:** Aeromedical retrieval services, Fixed-wing aircraft, OECD countries, Trauma and emergency retrieval

## Abstract

**Background:**

Aeromedical emergency retrieval services play an important role in supporting patients with critical and often life-threatening clinical conditions. Aeromedical retrieval services help to provide fast access to definitive care for critically ill patients in under-served regions. Typically, fixed-wing aeromedical retrieval becomes the most viable transport option compared with rotary-wing aircraft when distances away from centres of definitive care extend beyond 200 kms. To our knowledge, there are no studies that have investigated fixed-wing aeromedical services in the member countries of the organisation for economic cooperation and development (OECD). A description of the global characteristics of aeromedical services will inform international collaboration to optimise clinical outcomes for patients.

**Aim:**

In this scoping review, we aimed to describe the features of government- and not-for-profit organisation-owned fixed-wing aeromedical retrieval services in some of the member countries of the OECD.

**Methods:**

We followed scoping review methodology based on the grey literature search strategy identified in earlier studies. This mostly involved internet-based searches of the websites of fixed-wing aeromedical emergency retrieval services affiliated with the OECD member countries.

**Results:**

We identified 460 potentially relevant records after searching Google Scholar (n = 24) and Google search engines (n = 436). After removing ineligible and duplicate information, this scoping review identified 86 government-and not-for-profit-operated fixed-wing aeromedical retrieval services as existing in 17 OECD countries. Concentrations of the services were greatest in the USA followed by Australia, Canada, and the UK. The most prevalent business models used across the identified OECD member countries comprised the government, not-for-profit, and hybrid models. Three-quarters of the not-for-profit and two-fifths of the hybrid business models were in the USA compared to other countries studied. The government or state-funded business model was most common in Australia (11/24, 46%), Canada (4/24, 17%), and the UK (4/24, 17%). The frequently used service delivery models adopted for patients of all ages included primary/secondary retrievals, secondary retrievals only, and service specialisation models. Of these service models, primary/secondary retrieval involving the transportation of adults and children from community clinics and primary health care facilities to centres of definitive care comprised the core tasks performed by most of the aeromedical retrieval services studied. The service specialisation model provided an extra layer of specialist health care dedicated to the transportation of neonates and paediatrics. At least eight aeromedical retrieval services catered solely for children from birth to 16 years of age. One aeromedical service, the royal flying doctor service in Australia also provided primary health care and telehealth services in addition to primary retrieval and interhospital transfer of patients. The doctor and registered nurse/paramedic (Franco-German model) and the nurse and/or paramedic (Anglo-American model) configurations were the most common staffing models used across the aeromedical services studied.

**Conclusions:**

The development and composition of fixed-wing aeromedical emergency retrieval services operated by not-for-profit organisations and governments in the OECD countries showed diversity in terms of governance arrangements, services provided, and staffing models used. We do not fully understand the impact of these differences on the quality of service provision, including equitable service access, highlighting a need for further research.

**Supplementary Information:**

The online version contains supplementary material available at 10.1186/s13049-022-01053-x.

## Introduction

Aeromedical retrieval, also known as aero-medical evacuation, casevac, and medevac, was first used in World War I to quickly transport severely wounded soldiers from the battlefield to a medical facility [1, 2]. The role of aeromedical retrieval has expanded significantly over the past 100 years and includes fixed-wing and rotary-wing aircraft configured to provide medical services in locations where the ground ambulance is not a viable option [2–4]. Today, aeromedical retrieval medicine is the deployment of a specialised team of appropriately trained health care professionals in medically fitted aircraft to a patient’s location, often in rural and remote regions, to rescue and stabilise before transfer to definitive care in another location [3]. Aeromedical retrieval services bridge the gap in health care for communities in difficult-to-reach geographical locations with limited access to health care facilities [4, 5]. They provide a lifesaving alternative to a road ambulance service, in which some communities, often located in rural and remote areas, are unable to reach appropriate healthcare locally [6].

In systems where both rotary- and fixed-wing aircraft are available, the former are rarely used beyond an immediate emergency evacuation due to considerations mostly relating to service capabilities, operating costs, and the clinical needs of patients [7, 8]. Fixed-wing aeromedical retrieval becomes the most viable transport option compared with rotary-wing aircraft when distances away from centres of definitive care extend beyond 200 kms [9].

Aeromedical retrieval services typically comprise three types: primary retrieval, secondary retrieval, and tertiary retrieval [3]. Primary retrieval represents an emergency response to trauma or other medical emergencies in a prehospital location and most often results in the transportation of critically ill patients to a hospital for further treatment [13]. Secondary retrieval services include the provision of advanced care to patients in health care facilities with limited resources and expertise, for example, community clinics and primary health care centres. Tertiary retrieval, also known as inter-hospital transfer involves the movement of a patient from one specialised facility to another. Typically, tertiary retrieval involves the step-up transfer of patients between secondary and higher-level tertiary hospital care for more advanced ongoing clinical support. Tertiary retrieval may also involve the transfer of patients to less acute medical settings, also known as repatriations or back transfers, where health care is stepped down for ongoing support prior to medical discharge [4, 10].

Fixed-wing aeromedical retrieval services are an important component of emergency healthcare systems, enabling rapid response to medical emergencies, saving lives, and improving health outcomes. Despite this, no study has summarised and compared fixed-wing aeromedical retrieval services across the world. This study, therefore, maps and discusses fixed-wing aeromedical retrieval services operated by government and not-for-profit organisations across the world. We aimed to understand current organisational arrangements which could foster collaborations to improve health outcomes for communities disadvantaged by geographic location. We chose the government and not-for-profit fixed-wing aeromedical retrieval services due to the important role these services play in bringing emergency health care to disadvantaged communities in remote difficult-to-reach and often under-served regions.

### Objectives

Identify and describe fixed-wing aeromedical retrieval services operated by government and not-for-profit organisations of the member countries of the Organisation for Economic Cooperation and Development (OECD). This includes a description of the business, service delivery, and staffing models used by these aeromedical retrieval services.

### Research questions

This scoping review was guided by two research questions:

1. Which not-for-profit and government-funded fixed-wing aeromedical retrieval services exist in the OECD countries?
2. What business, service delivery, and staffing models are used by these aeromedical services?

### Study setting

**Fig. 1:**
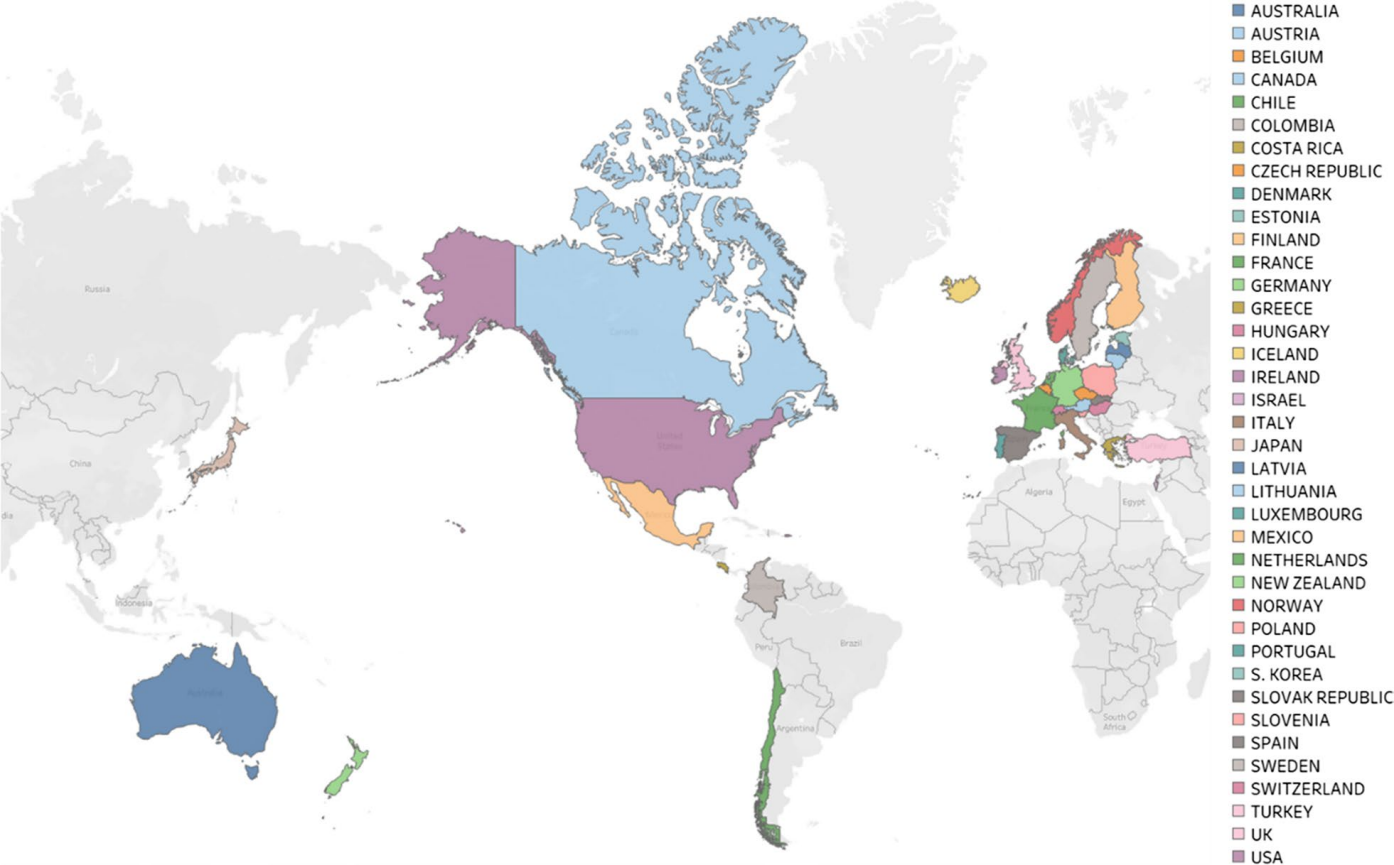
Map showing the 38 OECD countries

The study focused on fixed-wing aeromedical retrieval services located across the 38 member countries of the OECD (Fig. 1). We selected the OECD community because the global grouping includes countries that are located across most continents, are at different stages in their economic development, and have varied population densities and geographical footprints.

## Methods

This study was initially conceived as a systematic review. However, after the initial searches of peer-reviewed literature did not yield results of relevance for addressing our research questions and following consultations with an experienced librarian, we used the scoping review method to understand the breadth of fixed-wing aeromedical retrieval services in the OECD countries [11, 12]. We considered that a scoping review would be an appropriate tool to identify and map available information [13].

Due to the limited peer review literature, grey literature seemed to be the most likely source of information. We defined grey literature as referring to both published and unpublished information that is not peer-reviewed or indexed in bibliographic databases [14]. Grey literature includes government reports, technical papers, website notices, and other forms of non-academic documentation [15].

In the absence of a ‘gold standard’ tool for combing the grey literature, we adopted the strategy proposed by Godin et al. [15]. The methodological strategy involves four complementary stages: (a) search of grey literature databases (b) customised searches of the Google search engines, (c) search of targeted websites, and (d) consultation with experts in the field [15, 16]. This scoping review conforms to the PRISMA reporting standards [17, 18].

### Inclusion/exclusion criteria

We included articles, papers, and reports if they described, used, compared, or evaluated aeromedical retrieval services, as of 31 January 2022. We excluded the aeromedical retrieval services that were (a) operated solely by a for-profit organisation or company (b) owned by the armed forces (c) comprised rotary wing-based aircraft only (d) not operated in a country that is a member of the OECD. We narrowed the focus of the study in this way to avoid it becoming large and unwieldy. We also excluded literature or resources written in a language other than English due to translation issues.

### Search strategy

#### Grey literature databases

To identify fixed-wing aeromedical retrieval services in the OECD countries we performed searches of the peer-reviewed literature in the MEDLINE and Google Scholar databases. With expert guidance from the librarian we used the search terms: “air ambulanc”, “air ambulance retrieva”, “air ambulance servic”, “aeromedic* retrieva”, “aeromedic”, “aeromedic* evacuatio”, “fixed-wing air ambulanc”, “fixed-wing aeromedic”, “retrieval servic”, “scene response aeromedic”, “pre-hospital aeromedic” and “air medeva”.

#### Customised Google search engines

We next searched grey literature sources using the search engine Google. We used the same keywords as we did for the database search and added the search terms ‘fixed wing’, ‘aeromedical retrieval service’, and, ‘air ambulance’. We repeated the search 38 times including the ‘name of OECD country’.

#### Targeted websites

We searched the websites of some umbrella organisations for aeromedical retrieval services based on information accessed from Google search engines. This included the websites of the Association of Air Medical Services (AAMS), Aeromedical Society of Australasia (AST), the European Aero-Medical Institute (EURAMI), and the Commission on Accreditation of Medical Transport Systems (CAMTS). AAMS is a United States-based not-for-profit international trade association. It represents and advocates on behalf of the global air medical and critical care ground transport industry [19]. AST is a not-for-profit, membership-based organisation representing professionals and institutions involved with the provision of aeromedical services in Australia and New Zealand [20]. EURAMI is a not-for-profit organisation that accredits fixed-wing aeromedical services based in Europe. CAMTS is a United States-based not-for-profit organisation that offers voluntary accreditation services for member medical transport services internationally [21].

Searching websites continued until the end of January 2022. We stopped searching after it became apparent that additional searching of each website was unlikely to yield new information. This decision was adopted from the concept of ‘data saturation’ used in qualitative research [22, 23].

#### Consultation with experts in the field

Consistent with the approach suggested by Godin et al. [15, 16], we contacted 40 aeromedical retrieval services via email requesting information about their services. We sent reminder emails to addressees that did not respond to our earlier email. Of the 40 emails sent, we received only eight responses.

Several members of the research team (FG, SS, PKH, BS, PB, and ZS) are experts in aeromedical retrieval. During routine progress meetings, the team members contributed technical opinions and advice that informed the search strategy [15].

#### Study selection and data extraction

KM selected and screened information from the websites of the eligible aeromedical retrieval services based on the previously determined inclusion and exclusion criteria. Key information collected from the included data sources was compiled into an (Additional file 1). The information included: country of registration of the aeromedical service, name of aeromedical retrieval service, source of funding, type of the retrieval services offered (e.g., primary, secondary, or tertiary), the scope of the services provided, and the clinical make-up of the crew. The extracted data were independently checked by SW and MG. Ambiguities in data extraction were discussed until a consensus was reached.

### Quality of information

In this scoping review, we did not assess the quality of the information sourced through the grey literature searches. The procedure is consistent with the scoping review process described by other authors [12, 13].

### Data analysis

Consistent with the qualitative content analysis methodology suggested by Levac et al. [12], KM who was most familiar with the data, collated the search results to present a thematic summary of the findings. Themes and categories were initially determined ‘a priori’ from the data extraction headings (Additional file 1) and later renamed. Sub-themes were subsequently identified from the collected data. The preliminary themes and sub-themes were reviewed by all authors. Discrepancies were resolved through discussion. Unless otherwise specified in this review, aeromedical retrieval services refer to fixed-wing services.

## Results

### Literature searches

**Fig. 2:**
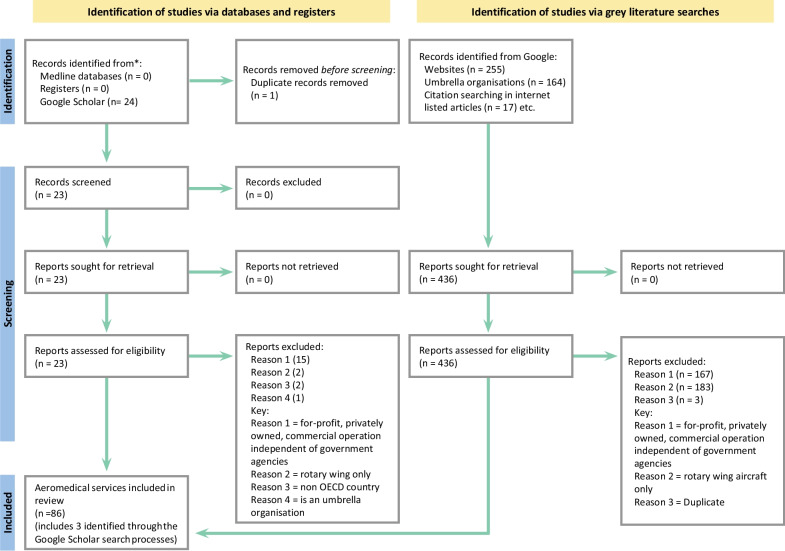
PRISMA Flowchart

The initial search process in MEDLINE did not yield any records of interest to address the research questions. The Google Scholar database identified 24 aeromedical retrieval services. The grey literature searches via Google search engine and targeted websites identified 436 potentially relevant information. After removing ineligible and duplicate information, only 86 fixed-wing aeromedical retrieval services were included in this study (Fig. 2: PRISMA Flowchart).

### Prevalence of aeromedical retrieval services in OECD countries

**Fig. 3:**
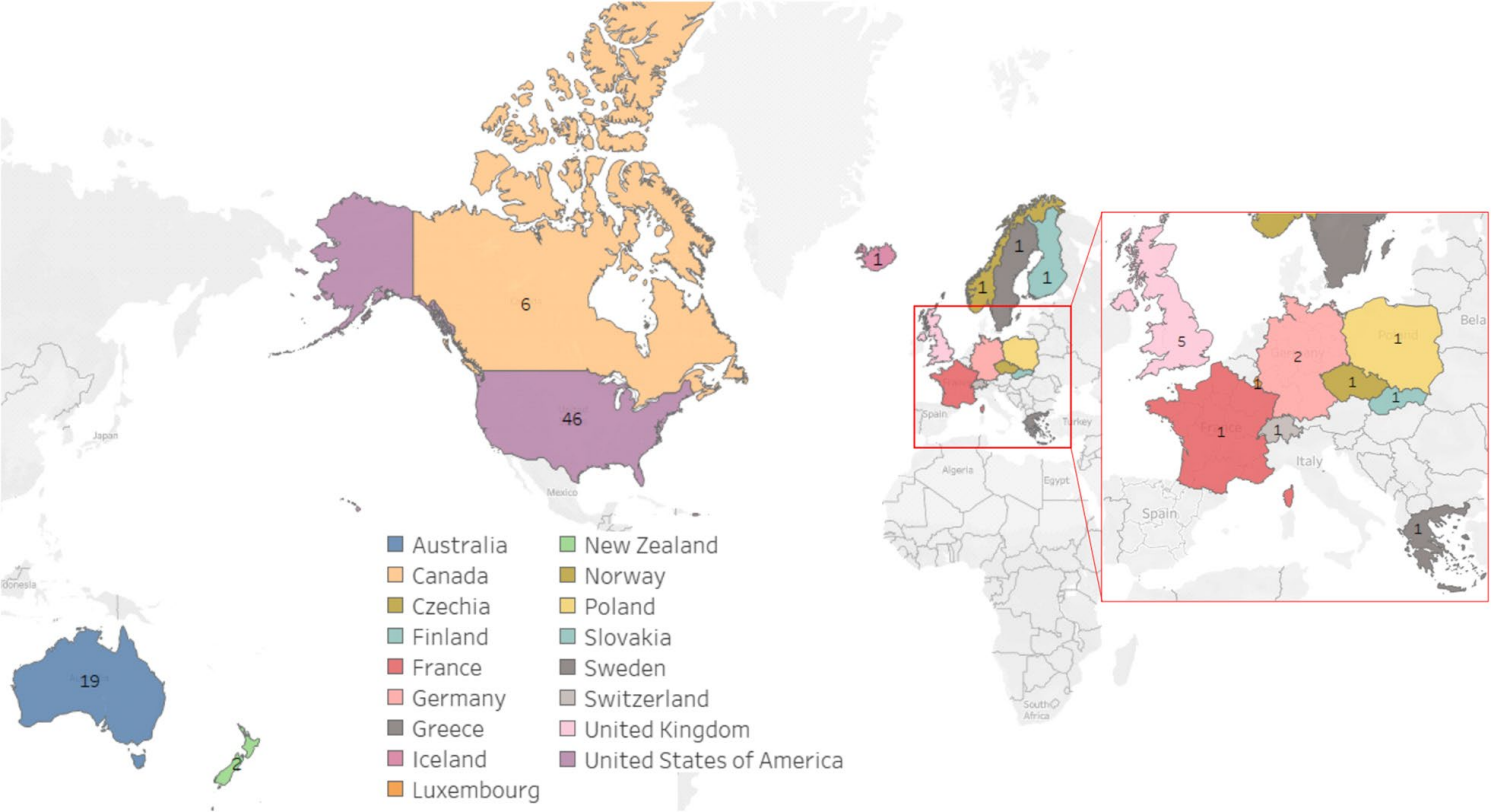
Map showing OECD countries with aeromedical emergency retrieval services

Of the 38 OECD member countries, we identified 17 countries with a total of 86 fixed-wing aeromedical retrieval services that were owned or operated by a not-for-profit or government organisation. The countries were Australia, Canada, Czechia, Finland, France, Germany, Greece, Iceland, Luxemburg, New Zealand, Norway, Poland, the Slovak Republic, Sweden, Switzerland, the United Kingdom (UK), and the United States of America (USA). The number of fixed-wing aeromedical retrieval services was the highest in the USA (46 aeromedical retrieval services), followed by Australia (n = 14), Canada (n = 6), and the UK (n = 5). Germany and New Zealand have two aeromedical services each and the remaining countries have one service each (Fig. 3 and Additional file 1: Table S1). The fixed-wing aeromedical retrieval services were located mostly in Europe, North America, and Australasia. OECD countries in South America (Chile, Colombia, Costa Rica, and Mexico) and Asia (Japan, South Korea) did not have any fixed-wing aeromedical retrieval services that were owned or operated by a government or not-for-profit organisation.

Fourteen of the 17 OECD countries with aeromedical retrieval services had a gross national income (GNI) above the 50th percentile, while five of the countries were below the 50th percentile for GNI. In this study, we define GNI as the total amount of money earned by a nation's people and businesses and includes the gross domestic product (GDP) and income from overseas remittances, property income, and net taxes minus payments out of the economy by businesses and investors based in other countries [24]. Countries with the largest land mass, the USA, Canada, and Australia had a greater proliferation of fixed-wing aeromedical retrieval services. We also observed that GNI and population size had no influence on the number of fixed-wing aeromedical retrieval services operated by a country. For instance, countries with high GNI such as Belgium, Denmark, and Ireland do not seem to have fixed-wing aeromedical services whilst other smaller high-income countries such as Luxembourg, and Switzerland had them. We also noted that countries with large geographical areas and high populations (e.g. Colombia, Mexico, Turkey) do not have fixed-wing aeromedical retrieval services (Table 1).

**Table 1:**
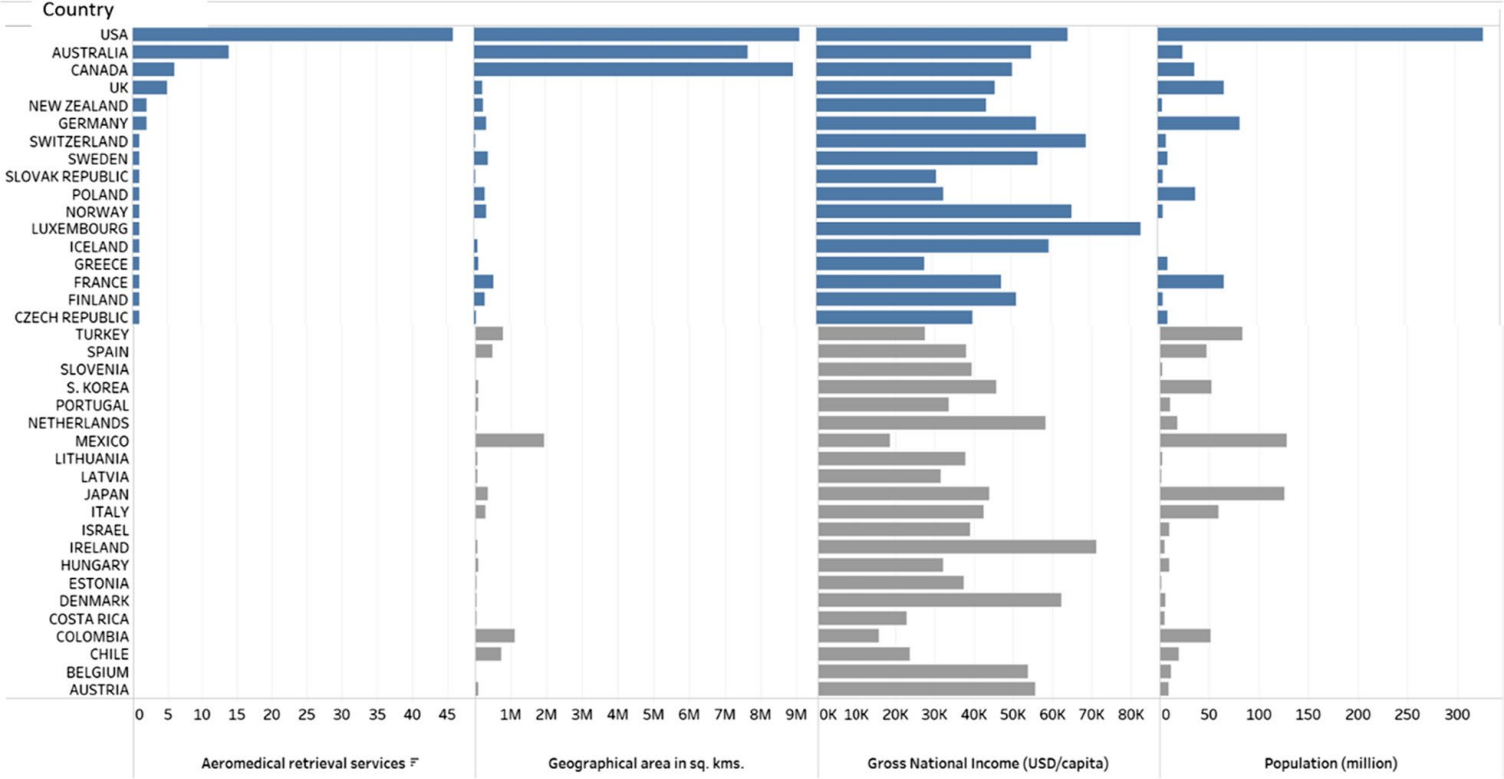
OECD countries with Gross national income, size of geographical area, population size, and number of aeromedical retrieval services

### Business models

We identified three business models that were predominantly used by the fixed-wing aeromedical retrieval services in the OECD countries studied: (a) government, (b) not-for-profit, and (c) hybrid [13, 14]. The not-for-profit organisation model was the most common business model used. The not-for-profit business model involved the provision of the medical staff, medical equipment, flight crews, medical services, and aircraft owned and operated by a not-for-profit organisation or a partnership of two or more not-for-profit organisations. We identified several fixed-wing aeromedical retrieval services that adopted the not-for-profit business model in Australia, Canada, Germany, Iceland, Luxembourg, Sweden, Switzerland, UK, and USA. With 28 not-for-profit fixed-wing aeromedical services, the USA had the highest number of such services of all the countries studied. Of the not-for-profit aeromedical services in the USA, a significant proportion was hospital-based. The not-for-profit business model was also observed in Australia (n = 3), Canada (n = 2), and Germany (n = 2).

The hybrid model was the second most frequently used business model. The hybrid model usually comprised the joint venture configurations of government/not-for-profit, government/for-profit, and not-for-profit/for-profit variations. Under the hybrid model, the not-for-profit or government bodies generally provide the medical staff and medical services whilst the for-profit organisation supplied the aircraft and flight crew [13, 14]. The government/not-for-profit business model was frequently encountered in Australia (n = 8). An example of a government/not-for-profit arrangement includes the collaborative arrangement bringing together the paediatrics and neonates service Neonatal Retrieval Service (NeoRESQ) in Australia with several government and not-for-profit organisations including Retrieval Services Queensland, Queensland Ambulance Service, Queensland Government Air, and the Royal Flying Doctor Service (RFDS) (Queensland Section) in which centralised aeromedical retrieval is coordinated by the Neonatology Department at the Royal Brisbane and Women’s Hospital (Additional file 1). The government/ for-profit business configuration was common in several European countries (n = 4) and Canada (n = 2) whilst the not-for-profit/for-profit business model was frequently observed in the USA (n = 19). In an example of a not-for-profit/for-profit arrangement, the Boston MedFlight aeromedical emergency retrieval service, a not-for-profit consortium of seven hospitals in Boston (USA), has its aircraft supplied and maintained by SevenBar Aviation, a for-profit entity.

The government model as a standalone business configuration was also common. This model typically involved the fixed-wing aeromedical retrieval services being provided by medical and flight staff, medical equipment, and aircraft employed, owned, or operated by a government organisation. Twenty-five fixed-wing aeromedical retrieval services operated on the government model which was most common in Australia (n = 11), Canada (n = 4), and the UK (n = 4).

### Funding models

The government-owned aeromedical retrieval services were typically publicly funded through grants. The not-for-profit aeromedical retrieval services were funded through a variety of funding sources including government grants, donations, corporate sponsorships, medical insurance, memberships, and fundraising. In the USA and Germany, health insurance and membership schemes comprise a key component of the funding models and healthcare system. Health insurance and membership cover aeromedical transport costs for individuals and their family members (spouse, children/dependents). Provincial health authorities and local governments (municipal authorities) also comprise an important source of funding for some aeromedical emergency retrieval services such as those found in Canada, New Zealand, and Sweden.

### Service delivery models

We identified three main service delivery models: primary/secondary retrieval combination, service specialisation, and the secondary retrieval-only model. The primary/secondary retrieval combination model was the most frequently used with 76 aeromedical retrieval services adopting it to support patients of all ages. The service specialisation model primarily focuses on the transportation of neonates and paediatrics. Twelve aeromedical retrieval services in the USA (n = 7), Australia (n = 2), the United Kingdom (n = 2), and Canada (n = 1) provide such specialist services. For example, the Newborn and Paediatric Emergency Transport Service in New South Wales and NeoRESQ in Queensland support neonates and paediatric patients only. Similarly, in the USA, the Children’s Mercy Critical Care Transport, the Paediatric AND Neonatal Doernbecher Transport, and the Neonatal and Paediatric Specialty Transport Services support neonatal and paediatric patients only. The ScotSTAR Neonatal Transport Service provides care and transportation of newborn infants in Scotland.

Three aeromedical services, two in the UK and one in the USA used the secondary retrieval-only model. Examples of the aeromedical retrieval services that adopted the secondary retrieval-only model include the Isle of Man Department of Health and Social Care and the Channel Islands of Guernsey and Jersey Air Ambulance.

Other less frequently used aeromedical retrieval service delivery models included primary health care services including outreach clinics, telehealth, and health promotion (RFDS), search and rescue (CareFlight, New South Wales Air Ambulance), organ transportation (New South Wales Air Ambulance, Ornge, CareFlite), and international retrieval services (CareFlight, LifeFlight Australia, Critical Care Transport).

We noted the existence of multi-country aeromedical service delivery models in which medical services were operated across coterminous national or state boundaries. For instance, Germany’s DRF Luftrettung operates in Germany, Austria, and Liechtenstein whilst the Babcock Scandinavian AirAmbulance of Sweden also operates under contract in Finland.

### Staffing models

We examined the staffing models used across the OECD countries included in this review. Whilst we noted that the typical flight medical crew comprised two health care providers, there was considerable variation between and amongst the fixed-wing aeromedical retrieval services studied. Some aeromedical services included a third medical team member. The most common skill mix configurations included the doctor and registered nurse or nurse-midwife (Australia, Greece, Norway, New Zealand, Luxemburg, and Finland) and doctor and paramedic (Germany, Israel, Iceland, France, and Poland), and doctor with a nurse or paramedic (Australia, New Zealand). Canada and the USA favoured a registered nurse and a paramedic crew whilst most aeromedical services in the United Kingdom were crewed by paramedics. For services specialising in paediatrics and neonates, their staff models were doctors with registered paediatric or neonatal nurses. Several aeromedical retrieval services had a medical crew that included a respiratory therapist and this was a common feature in the services that specialised in paediatrics and neonates.

It was rare for an aeromedical service to be staffed by only one health care provider or the nurse/nurse or paramedic/paramedic configurations. It was also rare (exceptions include the RFDS) for an on-call emergency physician to provide guidance to or accompany the medical team depending on patient issues. Generally, we observed that the staffing models varied widely depending on mission profile, local needs, and the prevailing aeromedical emergency retrieval policies.

Further investigation into staff qualifications showed that not all the identified aeromedical retrieval services provided this information on their websites. Those that did showed that the aeromedical retrieval staff are clinically experienced with advanced qualifications to ensure they can provide expert clinical support across many medical situations. We noted several variations in qualifications across the aeromedical retrieval services studied. Most registered nurses have experience in critical or intensive care units (ICU), or Emergency Departments (ED). Both registered nurses and paramedics usually held certificates in Basic Life Support (BLS), Basic Cardiac Life Support (BCLS), Advanced Cardiac Life Support (ACLS), Paediatric Advanced Life Support (PALS), Mobile Intensive Care, and Neonatal Resuscitation Program (NRP). Doctors were reported to hold qualifications in anaesthetics, emergency medicine, or intensive care. For example, the website of Life Flight Network (USA) reported that flight nurses were registered nurses trained in BLS, ACLS, PALS, NRP, Certified Emergency Nurse, Pre-Hospital Trauma Life Support (PHTLS), Transport Professional Advanced Trauma Course, plus 5 years ICU/ED experience and registered nurse licence. The flight paramedics held certifications in BLS, ACLS, PALS, NRP, PHTLS, and the Flight Paramedic Certificate in addition to the Paramedic certificate plus a minimum of 5 years of Emergency Medical Transport paramedic experience.

## Discussion

To our knowledge, this is the first scoping review of fixed-wing aeromedical retrieval services funded, owned, or operated by not-for-profit companies/organisations or national or state governments in the OECD member countries. This study focused on fixed-wing aeromedical retrieval services only and aimed to understand which countries have the aeromedical services and how they were structured in terms of business, funding, service delivery, and staffing models.

Looking at the OECD community provided a sample of member countries located on five continents that differed in population size, landscape, and range in GNI (Table 1) as well as the classification according to high- upper- or middle-income country [25]. Aeromedical retrieval is expensive and requires good infrastructure and as such, it may be expected that countries with these services are the wealthiest member countries. This did not seem to be the case for fixed-wing aeromedical services included in this scoping review. However, most OECD member countries are classed as high-income countries with a GNI per capita greater than USD 12,696 except Colombia, Costa Rica, Mexico, and Turkey (upper-middle-income, USD 4096–12,695) [25]. A small country with a good road network and health infrastructure seemed to have less need for fixed-wing aeromedical retrieval services than a country that is large with less infrastructure or a landscape that makes road travel arduous or impossible in inclement weather. Countries with the largest land mass; the USA, Canada, and Australia had a greater proliferation of fixed-wing aeromedical retrieval services but the next 3 largest countries, Colombia, Mexico, Turkey (upper-middle-income), and Chile (high income) had zero fixed-wing services. There was no discerning pattern for which country had a fixed-wing aeromedical retrieval service. Similarly, a review of the provision of helicopter emergency medical services (HEMS) in Europe, found no clear relationship between population size, land area, GDP, and HEMS [26]. Comparing those results to our results, 22 of the 26 European OECD countries have HEMS and 13 of those have both HEMS and fixed-wing services, one country (Iceland) has only fixed-wing service, whereas three countries (Estonia, Latvia, and Lithuania) have neither [26]. The distribution of HEMS in OECD countries was outside the scope of this review, but an area for further research would be to examine how the two types of aeromedical retrieval services complement each other, and the optimal mix to achieve the most cost-effective provision of aeromedical retrieval services.

Excluding for-profit only models, we identified three predominant business models including the not-for-profit, government, and hybrid models. We observed that the hybrid model comprised the public/for-profit, not-for-profit/for-profit, and not-for-profit/not-for-profit collaborations. The for-profit as a standalone model was not part of this scoping review. Collaborative arrangements involving resource-sharing and centralised coordination of aeromedical retrieval services were noted across several aeromedical services. Past studies suggested that the coordination of aeromedical retrieval services was linked with benefits including efficiency and effectiveness in service delivery and associated with reduced service fragmentation and the use of common protocols and procedures [9, 27]. Collaborative agreements make the most of existing resources providing an efficient way to establish an aeromedical retrieval service in a location beyond the range of existing services. When managed well collaborative arrangements can be very beneficial to the people they serve. However, many disparate services may provide a good emergency response but may fail to provide the complete health care a patient needs pre- and post- the emergency response.

Two predominant staffing models were identified: doctor and nurse/paramedic, and nurse and paramedic configurations. Using the multidisciplinary teams of health care providers may represent standard practice. Including midwives, paediatric nurses, neonatal nurses, obstetric nurses, and respiratory nurses can be perceived as adding to the standard team and enhancing access to specialist care [28]. The finding is consistent with the extant literature [29]. The doctor/physician-led configuration is often described as the Franco-German model in which the doctor/physician attends the patient at the scene of illness or injury before retrieval to centres of advanced care [30]. The nurse and the paramedic-led combination, also known as the Anglo-American model, involves the use of nurses and paramedics in a prehospital setting [30, 31]. Modified versions of the two predominant staffing models were also noted across most OECD countries studied. Presumably, the modifications were intended to fit the local context. Systematic reviews by Knapp et al*.* [32] and Laverty et al*.* [33] suggest the use of physician-led emergency medical teams or teams that have an advanced health care provider to improve mortality and reduce morbidity in the pre-hospital phase when the injury is serious. However, this model may come with a significantly higher financial cost [34]. Further studies to determine differences in patient outcomes and the costs involved in the hospital setting after arrival could inform aeromedical services on the best models to use.

Many services provided primary retrieval/secondary retrieval services. In Australia, interhospital transfers are far more frequent than primary retrievals [35]. A lack of tertiary and specialist facilities in rural and remote areas is the most obvious reason. A person can get life-saving intervention but then needs to be transferred to receive ongoing care. The tyranny of distance can make these journeys treacherous for patients which can be alleviated by aeromedical retrieval services [36].

## Strengths and limitations

This scoping review has relied on information accessed through internet-based searches of websites. Although the Internet provides an important source of information, it is well-established that the accuracy, comprehensiveness, and reliability of the information may not be accurate [37].

This scoping review adopted a multi-step search strategy. The approach may have helped to enhance the credibility and replicability of the searches although the online searching of websites may not have led to replicable results. Past studies have underscored the difficulty of achieving replicability when working with search engines, such as Google, whose search results can be distorted by recent popularity [38]. Furthermore, we are cognisant that governments and organisations often restrict access to their web pages through a process called geoblocking [39]. If a researcher from a different country did the same searches, they may access different results.

The search strategy involved sources written in English. Some OECD countries studied use languages other than English. Translation services were unavailable on some websites, and we could not do a manual translation. Therefore, these sources were excluded and could have contained relevant information.

Information gaps such as funding sources, size and make-up of the fixed-wing fleet, and the leading inflight diagnosis, made it difficult to fully explore our research questions. This has been an issue for previous studies that searched the grey literature [40]. The information gaps in this study made data synthesis challenging.

The scoping review did not include the rotary-wing aeromedical services. This may have reduced our understanding of the true size and composition of the aeromedical services available within the OECD member countries.

## Conclusions

This scoping review has identified fixed-wing aeromedical services operated in the OECD countries that are owned and operated by government or not-for-profit entities. We highlighted the development and composition of fixed-wing aeromedical retrieval services operated by not-for-profit organisations and governments in the OECD countries. We have identified the various business, funding, service delivery, and staffing models used. Based on the available information, we were unable to determine which models provide the best services. Further research to identify the gaps we have highlighted (Box 1) could provide valuable information on the cost-effectiveness, service delivery capability, and effectiveness of existing and new aeromedical retrieval services.

**Box 1:** Potential topics for further research

Areas for further research to determine the effectiveness of aeromedical retrieval services
Key Questions:
How equitable are aeromedical retrieval services for people living in countries where healthcare and aeromedical retrieval services are based on medical insurance or membership?
Which staffing models models provide better patient outcomes?
Do non-specialised retrieval services provide a suitable service for neonates and paediatric patients?
Can multi-country aeromedical services provide a good level of care whilst reducing administrative costs for smaller countries?
How do fixed-wing and rotor-wing services services complement each other to achieve the most cost effective aeromedical retrieval service?

## Supplementary Information

**Additional file 1**. Table S1.

## Data Availability

The datasets analysed during the current scoping review are available from the corresponding author upon reasonable request.

## Abbreviations

AAMS: Association of air medical services
ACLS: Advanced cardiac life support
AST: Aeromedical society of Australasia
BCLS: Basic cardiac life support
BLS: Basic life support
CAMTS: Commission on accreditation of medical transport systems
ED: Emergency department
EURAMI: European aero-medical institute
GDP: Gross domestic product
GNI: Gross national income
HEMS: Helicopter emergency medical services
ICU: Intensive care unit
NeoRESQ: Neonatal retrieval service
NRP: Neonatal resuscitation program
OECD: Organisation for economic cooperation and development
PALS: Paediatric advanced life support
PHTLS: Pre-hospital trauma life support
RFDS: Royal flying doctor service
UK: United Kingdom
USA: United States of America
