# Supplementary material for: Aeromedical retrieval services characteristics globally: a scoping review
Source: Scand J Trauma Resusc Emerg Med. 2022 Dec 12;30:71. doi: 10.1186/s13049-022-01053-x (PMC9743498; doi:10.1186/s13049-022-01053-x)
Supplement: Supplementary file 1 — Additional file 1. Table S1. [file 13049_2022_1053_MOESM1_ESM.docx]

**Additional File 1**

**Aeromedical retrieval services characteristics: a scoping review**

| **Country of registration** | **Name of aeromedical service** | **Source of funding** | **Type of fleet** | **Type of service** | **Clinical make-up of crew** | **Other relevant information** |
| --- | --- | --- | --- | --- | --- | --- |
| Australia | Royal Flying Doctor Service (RFDS)  Ambulance Victoria (Air Ambulance)  Adult Retrieval Victoria (ARV)  MedSTAR (established 2009) South Australia  Retrieval Service Queensland (RSQ)  Ambulance Service of NSW (ASNSW)  Newborn and paediatric Emergency Transport Service (NETS). NSW and Tasmania  NSW Aeromedical and Medical Retrieval Service (AMRS).  Aeromedical and Medical Retrieval Tasmania  Neonatal Retrieval Service (NeoRESQ), Queensland  CareFlight  LifeFlight Australia  Queensland Government Air (QGAir)  New South Wales Air Ambulance  Newborn Emergency Transport Service of Western Australia (NETS WA) | not-for-profit (donations, government grants, corporate sponsors, fundraising).  government funded  government funded  government funded  government funded.  government funded  government funded  government funded  government funded  not for profit (donations, government grants, corporate sponsors, fundraising).  not for profit (donations, government grants, corporate sponsors, fundraising).  government funded  government funded  government funded | fixed-wing and rotary wing  fixed-wing and rotary wing  fixed-wing (contracted from RFDS and private jet charter) and rotary wing  fixed-wing and rotary wing  fixed-wing and rotary wing  fixed-wing and rotary wing  fixed-wing and rotary wing  fixed-wing aircraft, occasionally rotary wing  fixed-wing and rotary wing  fixed-wing and rotary wing  fixed-wing and rotary wing  fixed-wing and rotary wing  fixed and rotary wing  fixed wing | primary and secondary retrieval of children (including neonates and paediatrics) and adults. Also, they provide fly-in primary health care services including telehealth  primary and secondary retrieval  primary and secondary retrieval of children from birth to 16 years of age and obstetric emergencies  primary and secondary retrieval  primary and secondary retrieval  primary and secondary retrieval  primary and secondary retrieval    primary and secondary retrieval  primary and secondary retrieval  primary and secondary retrieval, search and rescue. Also provide an international service  primary and secondary retrieval  primary and secondary including organ retrieval, search and rescue  primary and secondary  primary, secondary and tertiary | doctor and registered nurse, allied health, paramedic  doctor, registered nurse or paramedic  doctor, registered nurse or paramedic  doctor, registered nurse or paramedic  registered nurse and midwife with critical care experience; emergency medicine, intensive care or coronary care.  doctor, registered nurse or paramedic  doctor, registered nurse or paramedic  doctor, registered nurse or paramedic  doctor and registered nurse  doctor and registered nurse  doctor, registered nurse or paramedic  doctor and registered nurse or paramedic  doctor and registered nurse or paramedic  neonatal-trained doctors & nurses | 7 entities under a federated structure and working together under a joint venture agreement. Each of the entities is independent, both financially and operationally, with their Board and management. Each entity is a charity, registered with and regulated by the Australian Charities and Not-for-profit Commission. Flight nurse: Registered Nurse/midwife, medical practitioners, allied health, intensive care paramedic, critical care nurse. Doctors trained in anaesthetics, emergency, intensive care. The flight nurses are registered-nurse qualified with critical care experience and postgraduate certification training. Midwifery, neonatal and paediatric experience. As well as retrieval training  The service reaches across Victoria and into parts of southern New South Wales, northern Tasmania and parts of South Australia. Provides inter-hospital transfer of critically ill adult patients across the state through Adult Retrieval Victoria (ARV). Paramedics have a recognised Paramedicine 3 -year university degree qualification. Also training in Advanced Life Support (ALS) and Mobile Intensive Care (MIC) and graduate paramedics who have completed recognised university degrees. cs.  https://www.ambulance.vic.gov.au/about-us/our-services/patient-transport/  SAAS MedSTAR is South Australia’s single emergency medical retrieval service. Provides emergency transport between hospitals for the critically ill and injured, rapid response for pre-hospital trauma care and specialist escorted inter-hospital transfers including national and international aeromedical retrievals and repatriation. SAAS MedSTAR also performs back transfers of patients to rural and interstate facilities for paediatric and neonatal patients on a regular basis. For fixed wing aircraft, MedSTAR contracts the Royal Flying Doctor Service or jet charter). Also conduct national and international aeromedical retrievals and repatriation and support to South Australian health practitioners  RSQ caters as the nerve centre for emergency rescue operations that require helicopters or fixed-wing aircraft to get patients to the Queensland's hospitals. RSQ coordinates the tasking of aeromedical retrieval and inter-hospital transport of patients across the whole of Queensland between government and non-government providers including Queensland Government Air (QGAir), Queensland Ambulance Service, Royal Flying Doctor Service, LifeFlight Queensland, CQ Rescue (RACQ), Capricorn Helicopter Rescue (RACQ), Babcock Mission Critical Services. Provides adult, neonatal, paediatric, and high risk obstetric clinical coordination services with ten rotary and seven fixed-wing aircraft bases.  NSW Ambulance is an agency of NSW Health and the statutory provider of pre-hospital emergency care and ambulance services in the state of New South Wales, Australia. Collaborates with CareFlight and the Royal Flying Doctor Service work with NSW Ambulance to provide services in certain NSW locations. Flight nurse are all registered nurse and midwives with critical care experience, emergency medicine, intensive care or coronary care.  NETS' is the state-wide emergency service for medical retrieval of critically ill newborns, infants and children in New South Wales (NSW), Australia. It is part of NSW Ambulance. Registered nurse perform patient inter-hospital transfers and retrievals and possess experience in a Level 3 or 4 Special Care Nursery, neonatal and or paediatric intensive care or a retrieval service. Doctors will have experience in neonatal and/or paediatric critical care.  Air Ambulance, the fixed wing service of the Ambulance Service of NSW (ASNSW) located at Sydney Airport, Mascot. The aircraft, pilot and ground support staff are supplied by the Royal Flying Doctor Service, whilst the clinical staff are from ASNSW. Trauma retrieval and inter hospital transfer coordinated by a central Aeromedical Operations Centre of the Ambulance Service of NSW (ASNSW) in Sydney.  Aeromedical and Medical Retrieval Tasmania is a division of Ambulance Tasmania (AT) Aeromedical and Retrieval, Ambulance Tasmania is the single provider of adult aero-medical retrieval for the state of Tasmania. Provides inter-facility adult and paediatric retrievals. Fixed-Wing component of AMR is operated in partnership with the RFDS, providing interfacility transport, critical care support to rural and remote regions including the Bass Strait Islands, and interstate retrieval.  NeoRESQ is the neonatal retrieval service that supports transportation of premature or critically ill babies to life-saving treatment at regional centres in central and south east Queensland and northern New South Wales. Services are provided in partnership with Retrieval Services Queensland, Queensland Ambulance Service, Life Flight, Queensland Government Air and the Royal Flying Doctors Service. The centralised retrieval service is coordinated at the Royal Brisbane and Women’s Hospital (RBWH) Neonatology Department with neonatal retrievals being undertaken by staff from both the RBWH and Mater Mothers’ Hospital. Staff includes doctors and nurse who specialise in caring for newborn, sick and preterm infants.  CareFlight is a charitable organisation contracted to provide air ambulance services by the NT and NSW State governments. Also funded by philanthropic organisations, donations. Service international locations including Norfolk Island, New Zealand, Asia, Micronesia and Pacific regions. Nurse are intensive care, emergency department and aviation nursing trained. Doctors trained in emergency medicine, intensive care or anaesthetics.  <https://careflight.org/about-us/history/>  LifeFlight operates operate on a not-for-profit basis, supported by State Government funding, community donations and corporate sponsorships. Provides air ambulance to service the needs of the Queensland Health Department. Caters for international transfers covering the Asia-Pacific region. Doctors trained in critical care and nurse trained in intensive care. Contracted by Queensland Health to provide its air-medical staff across the state’s aeromedical network.  Weblink: <https://www.lifeflight.org.au/purpose-and-history/>  Represents a merger of Emergency Management Queensland Helicopter Rescue, Government Air Wing and Police Air Wing. In April 2021 the functions and staff of Queensland Government Air (QGAir) transferred to the Queensland Police Service (QPS). Queensland Government Air (QGAir) delivers lifesaving, community safety and state support aviation services to the people and government of Queensland.  https://www.psba.qld.gov.au/services/qgair/Pages/default.aspx  Rex subsidiary Pel-Air Aviation is contracted to provide air ambulance services to the NSW Ambulance Air Ambulance. NSW Ambulance collaborates with CareFlight and the Royal Flying Doctor Service to provide health care services in certain NSW locations. The Aeromedical Control Centre is staffed by a multi-disciplinary team of paramedics, critical care nurses and senior critical care doctors who triage patients.  https://www.ambulance.nsw.gov.au/our-services/operation  https://www.ambulance.nsw.gov.au/our-services/operations  The Newborn Emergency Transport Service (NETS WA) is a mobile intensive care unit for sick newborn and young infants who need expert neonatal care units that are available at King Edward Memorial Hospital or Perth Children’s Hospital. NETSWA collaborates with St John Ambulance, Royal Flying Doctor Service (RFDS) and Medical Air. <https://cahs.health.wa.gov.au/Our-services/Neonatology/Newborn-Emergency-Transport-Service> |
| Canada | British Columbia Ambulance Service (BCAS)  Ambulance New Brunswick's Air Ambulance - often referred to as AirCare  EHS LifeFlight (Nova Scotia)  Ornge (formerly Ontario Air Ambulance Corporation)  Saskatchewan Air Ambulance (also called LIFEGUARD)  ACCESS (Aeromedical Critical Care Emergency Services Specialists) | government funded  government funded  government funded  not for profit (donations, government grants, commercial insurance, medical insurance, private payers, corporate sponsors, fundraising).  not for profit (donations, government grants, commercial insurance, medical insurance, private payers, corporate sponsors, fundraising).  government funded | fixed-wing and rotary wing  fixed-wing and rotary wing  fixed-wing and rotary wing  fixed-wing and rotary wing  fixed-wing aircraft only  fixed-wing | primary and secondary retrieval of adults, paediatric, neo-natal and high-risk obstetrics patients.  primary and secondary retrieval  primary and secondary retrieval of adult, neonatal, paediatric and obstetric patients  primary and secondary and organ transportation  primary and secondary retrieval  primary and secondary retrieval | registered nurse and paramedic  registered nurse and paramedic  registered nurse & paramedic, paediatric/neonatal care, respiratory therapist, obstetric registered nurse  registered nurse, respiratory therapist or paramedic  registered nurse or paramedic  registered nurse and/or paramedic | Funded by British Columbia Emergency Health Services through the Provincial Health Services Authority (PHSA). Registered nurses are trained in Basic Cardiac Life Support, Advanced Cardiac Life Support, Paediatric Advanced  Life Support, and the Trauma Nursing Core Course. They also receive additional training including aircraft safety, advanced airway management (which includes mechanical ventilation and endotracheal intubation), flight. physiology/barophysiology, as well as land and water survival. Paramedics are Advanced Care Paramedics trained.  http://www.bcehs.ca/about/who-we-are/bc-ambulance-service  ANB is funded by the Government of New Brunswick. ANB is responsible for providing land and air ambulance services throughout New Brunswick.  Weblink: https://ambulancenb.ca/en/what-we-do/services/  LifeFlight is delivered under contract to the Government of Nova Scotia's Department of Health by Emergency Medical Care Inc. (EMC). EMC provides medical staff however the operation of the aircraft is sub-contracted to PAL Aerospace Limited.  Weblink: <https://novascotia.ca/dhw/ehs/EHS-system.asp>  <https://ehslifeflight.ca/about-us/>  Ornge (formerly Ontario Air Ambulance Corporation) is a Canadian not-for-profit corporation and registered charity that provides air ambulance services for the province of Ontario, under the direction of the province's Ministry of Health. Ornge (formerly the Ontario Air Ambulance Services Co.) coordinates all aspects of Ontario’s air ambulance system, including the contracting of flight service providers, medical oversight of all air paramedics, air dispatch, and authorising air and land ambulance transfers.  Weblink: <https://www.ornge.ca/about>  Saskatchewan Air Ambulance (also called LIFEGUARD) is the air ambulance service for the province of Saskatchewan based in Saskatoon and is owned and managed by Saskatchewan’s Ministry of Central Services. It also coordinates with Shock Trauma Air Rescue Society (STARS). STARS is a non-profit organization that provides air medical transportation for critically ill and injured patients by helicopter.  ACCESS partners with the Government of NWT Health and Social Services to provide emergency to 33 communities in the North West Territories. Medical crews provided through adult health service whilst aircraft and flight crews are provided by Air Tindi based in Yellowknife and Aklak Air based in Inuvik. |
| Czech Republic | Air Transport Europe Medical Air Transport - ATE | public/private partnership | fixed and rotary wing | primary and secondary retrieval | doctor | Air Transport Europe Medical Air Transport - ATE currently provides Helicopter Emergency Medical Service as a private healthcare provider based on the license granted by the Ministry of Health of the Czech Republic.  https://www.ate.sk/en/about/medical-transport/ |
| Slovak Republic | Air Transport Europe Medical Air Transport - ATE | public/private partnership | fixed and rotary wing | primary and secondary retrieval | doctor | Air Transport Europe Medical Air Transport - ATE currently provides Helicopter Emergency Medical Service as a private healthcare provider based on the license granted by the Ministry of Health of the Slovak Republic.  https://www.ate.sk/en/about/medical-transport/ |
| Finland | Babcock Scandinavian AirAmbulance | public/private partnership | fixed and rotary wing | primary and secondary retrieval | doctor and registered nurse | Babcock Scandinavian AirAmbulance is a Swedish helicopter and fixed-wing airline that operates primarily air ambulance helicopters and planes for the Finnish and Norwegian governments under contract and service county councils and municipalities, Babcock Scandinavian AirAmbulance is responsible for all fixed wing air ambulance operations in Norway, All medical personnel on board will be employed by Norway’s public health service. Nurses have specialist competence in anaesthesia intensive care and aviation medicine and at least five years of clinical experience.http://www.airamb.se/om-foretaget/om-foretagethttps://babcockinternational.fi/babcock-suomi/ |
| Norway | Air Ambulance Service of Norway  [Luftambulansetjenesten ANS" (LABAS) ] | government funded | fixed and rotary wing | primary and secondary retrieval | doctor and registered nurse | Air Ambulance Services of Norway is responsible for all air ambulance (both fixed wing aircraft and helicopters) in Norway. The publicly funded service is part of the national prehospital chain of care and is owned by the four regional health authorities (RHF) in Norway. All operations of the air ambulances in Norway are paid for and fully financed by the public sector. Two civilian air operators are under contract by Air Ambulance Services of Norway; Babcock Scandinavian AirAmbulance AS: provides all the fixed wing aircraft. Norsk Luftambulanse is fully owned by the Norwegian Air Ambulance Foundation. Babcock Scandinavian AirAmbulance is a Swedish helicopter and fixed-wing airline that operates primarily air ambulance helicopters and planes for the Swedish, Finnish and Norwegian governments. Registered nurses have a basic education in anesthesia and/or intensive care and special education in flight medicine.https://www-luftambulanse-no.translate.goog/ |
| Sweden | Kommunalförbundet Svenskt Ambulansflyg” (KSA) | not for profit, publicly funded | fixed and rotary wing | primary and secondary retrieval | doctor and registered nurse | Swedish Ambulance Flight (KSA) took over from Babcock Scandinavian Air Ambulance in 2021. Babcock Scandinavian AirAmbulance is a Swedish helicopter and fixed-wing airline that operated air ambulance helicopters and planes for the Swedish, Finnish and Norwegian governments. Kommunalförbundet Svenskt Ambulansflyg (KSA) is a municipal association with a federal council and federal board and is owned and financed by all of Sweden's 21 regions.  https://www.svenskt-ambulansflyg.se/om-ksa/ |
| France | EmService d'Aide Médicale Urgente (Urgent Medical Aid Service) [SAMU]. | government funded based on contracts with commercial companies | fixed and rotary wing | primary and secondary retrieval | doctor, registered nurse and /or a SMUR emergency technician. | The main ambulance services in France are provided by the SAMU – Services d’Aide Médicale Urgente, which is part of the public hospital system. There are around 100 SAMU call centres in France each run by the local major hospital. The service is organized based on the departments of France. Each department has a hospital-based SAMU organisation which is named with the department's unique two-digit number code. Each service is provided by a commercial contractor and may cover several departments.https://www.french-property.com/guides/france/public-services/health/accident-emergency/ |
| Germany | ADAC Luftrettung gGmbH  DRF Luftrettung | not for profit (donations, government grants, commercial insurance, medical insurance, private payers, corporate sponsors, fundraising).  not for profit (donations, government grants, commercial insurance, medical insurance, private payers, corporate sponsors, fundraising). | fixed and rotary wing  fixed and rotary wing | primary and secondary retrieval  primary and secondary retrieval | doctor and a paramedic.  doctor and a paramedic. | The Ministry of the Interior operates the fleet via its charitable subsidiary ADAC Luftrettung gGmbH,  The DRF Luftrettung (formerly Deutsche Rettungsflugwacht e.V.) is part of the German emergency medical services as a branch of the air medical services. The DRF Luftrettung consists of an independent, non-profit association, a foundation under civil law and a non-profit company. The DRF Foundation Air Rescue non-profit AG is one hundred percent owned by the DRF Foundation Air Rescue. The DRF Luftrettung and its subsidiaries operate in Germany, Austria, and Liechtenstein. |
| Greece | Ministry of Health | charitable organisations, government funded | fixed and rotary wing | primary and secondary retrieval | doctor and registered nurse | In Greece, pre-hospital emergency care services (PEMS) are an independent public institution. Fixed wing aircraft are owned by the Ministry of Health managed through the National Center for Emergency Care (EKAV) and operated by the Hellenic Air Force. |
| Iceland | Myflug air | not for profit (donations, government grants, commercial insurance, medical insurance, private payers, corporate sponsors, fundraising). | fixed-wing aircraft | primary and secondary retrieval | doctor and paramedic | Mýflug Air provides air ambulance services to a large part of Iceland under a contract with the Icelandic Ministry of Health and is operated in cooperation with The Akureyri Fire Department and the local hospital. |
| Luxembourg | LAR-Luxembourg Air Rescue service | not for profit (donations, government grants, commercial insurance, medical insurance, private payers, corporate sponsors, fundraising). | fixed and rotary wing | primary and secondary retrieval | doctor and registered nurse | Founded in 1988 as a non-profit organization, Luxembourg Air Rescue (LAR) |
| New Zealand | New Zealand Air Ambulance Service (NZAAS)  Air Ambulance Service | government funded  government funded. | fixed and rotary wing  fixed and rotary wing | primary and secondary retrieval  primary and secondary retrieval | doctor, paramedic and registered nurse  doctor and paramedic or registered nurse | NZAAS and Skyline Aviation are one of the largest aeromedical service in the country and hold contracts with District Health Boards (DHBs), Accident Compensation Corporation (ACC) and the Ministry of Health (MoH).  https://www.nzaas.co.nz/what-we-do/national-fixed-wing-retrieval/  Aircraft contracted from commercial operators, Life Flight, Philips Search & Rescue Trust (PSRT), Skyline Aviation Ltd, GCH Aviation and HeliOtago by the Ministry of Health, ACC and the various DHBs of the Northern, Central and Southern regions.  https://www.health.govt.nz/new-zealand-health-system/key-health-sector-organisations-and-people/national-ambulance-sector-office-naso/ambulance-services-quality-and-safety |
| Poland | Polish Lotnicze Pogotowie Ratunkowe (LPR) | government funded | fixed and rotary wing | primary and secondary retrieval | doctor and paramedic | The Polish Medical Air Rescue was established in May 2000 and financed by the Ministry of Health. Registered nurses must have at least secondary education as a nurse or paramedic and at least three years of experience working as a paramedic/nurse.  https://www.lpr.com.pl/en/about-us/history/ |
| Switzerland | Swiss Air Rescue REGA | not for profit, donations, fundraising | fixed and rotary wing | primary and secondary retrieval | doctor and registered nurse or paramedic | Rega is an independent, humanitarian and charitable foundation. Rega provides its services without public subsidies.  https://www.swiss-air-ambulance.ch/  https://www.rega.ch/en/ |
| USA | Life Flight Network  Lifeguard Air Emergency Services  Mercy Flight  MedCenter Air  North Flight Aero Med  REMSA Care Flight  AIRLIFE Denver  Airlift Northwest Air Medical Service  IU Health LifeLine  Grace on Wings  CareFlite  Critical Care Transport  Air St Luke’s Medical Transport  Carolina Air Care  Children's One Emergency Transport  HELP (Helicopter Emergency Lifesaving Program) Flight  Paediatric And Neonatal Doernbecher Transport (PANDA)  Neonatal and Paediatric Specialty Transport Services  Sanford AirMed  Vanderbilt LifeFlight  STAT MedEvac  Avera Careflight Emergency Air Transport  Cook Children's Transport Services  Paediatric Critical Care Transport  Memorial Hermann, Life Flight  Boston Children's Transport Program  Boston MedFlight  AirMed  Intermountain Life Flight  Survival Flight  Life Link III  Mayo Clinic Ambulance Service  Children's Mercy Critical Care Transport (CMCCT)  Benefis Mercy Flight  Air Link  West Michigan AirCare  Memorial MedFlight  San Juan Regional AirCare  Angel One Patient Transport System  CareFlight of the Rockies  Flight For Life Colorado  Cleveland Clinic's Critical Care Transport (CCT)  Nationwide Children’s Hospital's Critical Care Transport  LifeFlight® Critical Care Transport  Metro Life Flight  SCL Health  Regional West Air Link | not for profit (donations, government grants, commercial insurance, medical insurance, private payers, corporate sponsors, fundraising)  not for profit (donations, government grants, commercial insurance, medical insurance, private payers, corporate sponsors, fundraising)  not for profit (donations, government grants, commercial insurance, medical insurance, private payers, corporate sponsors, fundraising)  not for profit (donations, government grants, commercial insurance, medical insurance, private payers, corporate sponsors, fundraising)  not for profit (donations, government grants, commercial insurance, medical insurance, private payers, corporate sponsors, fundraising)  not for profit (donations, government grants, commercial insurance, medical insurance, private payers, corporate sponsors, fundraising)  not for profit (donations, government grants, commercial insurance, medical insurance, private payers, corporate sponsors, fundraising)  not for profit (donations, government grants, commercial insurance, medical insurance, private payers, corporate sponsors, fundraising)  not for profit (donations, government grants, commercial insurance, medical insurance, private payers, corporate sponsors, fundraising)  not for profit (donations, government grants, commercial insurance, medical insurance, private payers, corporate sponsors, fundraising)  not for profit (donations, government grants, commercial insurance, medical insurance, private payers, corporate sponsors, fundraising)  not for profit (donations, government grants, commercial insurance, medical insurance, private payers, corporate sponsors, fundraising)  not for profit (donations, government grants, commercial insurance, medical insurance, private payers, corporate sponsors, fundraising)  not for profit (donations, government grants, commercial insurance, medical insurance, private payers, corporate sponsors, fundraising)  not for profit (donations, government grants, commercial insurance, medical insurance, private payers, corporate sponsors, fundraising)  not for profit (donations, government grants, commercial insurance, medical insurance, private payers, corporate sponsors, fundraising)  not for profit (donations, government grants, commercial insurance, medical insurance, private payers, corporate sponsors, fundraising)  not for profit (donations, government grants, commercial insurance, medical insurance, private payers, corporate sponsors, fundraising)  not for profit (donations, government grants, commercial insurance, medical insurance, private payers, corporate sponsors, fundraising)  not for profit (donations, government grants, commercial insurance, medical insurance, private payers, corporate sponsors, fundraising)  not for profit (donations, government grants, commercial insurance, medical insurance, private payers, corporate sponsors, fundraising)  not for profit (donations, government grants, commercial insurance, medical insurance, private payers, corporate sponsors, fundraising)  not for profit (donations, government grants, commercial insurance, medical insurance, private payers, corporate sponsors, fundraising)  not for profit (donations, government grants, commercial insurance, medical insurance, private payers, corporate sponsors, fundraising)  not for profit (donations, government grants, commercial insurance, medical insurance, private payers, corporate sponsors, fundraising)  not for profit (donations, government grants, commercial insurance, medical insurance, private payers, corporate sponsors, fundraising)  not for profit (donations, government grants, commercial insurance, medical insurance, private payers, corporate sponsors, fundraising).  not for profit (donations, government grants, commercial insurance, medical insurance, private payers, corporate sponsors, fundraising)  not for profit (donations, government grants, commercial insurance, medical insurance, private payers, corporate sponsors, fundraising)  not for profit (donations, government grants, commercial insurance, medical insurance, private payers, corporate sponsors, fundraising)  not for profit (donations, government grants, commercial insurance, medical insurance, private payers, corporate sponsors, fundraising)  not for profit (donations, government grants, commercial insurance, medical insurance, private payers, corporate sponsors, fundraising)  not for profit (donations, government grants, commercial insurance, medical insurance, private payers, corporate sponsors, fundraising)  not for profit (donations, government grants, commercial insurance, medical insurance, private payers, corporate sponsors, fundraising)  not for profit (donations, government grants, commercial insurance, medical insurance, private payers, corporate sponsors, fundraising)  not for profit (donations, government grants, commercial insurance, medical insurance, private payers, corporate sponsors, fundraising)  not for profit (donations, government grants, commercial insurance, medical insurance, private payers, corporate sponsors, fundraising)  not for profit (donations, government grants, commercial insurance, medical insurance, private payers, corporate sponsors, fundraising)  not for profit (donations, government grants, commercial insurance, medical insurance, private payers, corporate sponsors, fundraising)  not for profit (donations, government grants, commercial insurance, medical insurance, private payers, corporate sponsors, fundraising)  not for profit (donations, government grants, commercial insurance, medical insurance, private payers, corporate sponsors, fundraising)  not for profit (donations, government grants, commercial insurance, medical insurance, private payers, corporate sponsors, fundraising)  not for profit (donations, government grants, commercial insurance, medical insurance, private payers, corporate sponsors, fundraising)  not for profit (donations, government grants, commercial insurance, medical insurance, private payers, corporate sponsors, fundraising)  not for profit (donations, government grants, commercial insurance, medical insurance, private payers, corporate sponsors, fundraising)  not for profit (donations, government grants, commercial insurance, medical insurance, private payers, corporate sponsors, fundraising)  not for profit (donations, government grants, commercial insurance, medical insurance, private payers, corporate sponsors, fundraising) | fixed and rotary wing  fixed and rotary wing  fixed and rotary wing  fixed and rotary wing  fixed and rotary wing  fixed and rotary wing  fixed and rotary wing  fixed and rotary wing  fixed and rotary wing  fixed and rotary wing  fixed-wing  fixed and rotary wing  fixed and rotary wing  fixed and rotary wing  fixed and rotary wing  fixed and rotary wing  fixed and rotary wing  fixed and rotary wing  fixed and rotary wing  fixed and rotary wing  fixed and rotary wing  fixed and rotary wing  fixed and rotary wing  fixed and rotary wing  fixed and rotary wing  fixed and rotary wing  fixed and rotary wing  fixed and rotary wing  fixed and rotary wing  fixed and rotary wing  fixed and rotary wing  fixed and rotary wing  fixed and rotary wing  fixed and rotary wing  fixed and rotary wing  fixed and rotary wing  fixed-wing  fixed and rotary wing  fixed and rotary wing  fixed and rotary wing  fixed and rotary wing  fixed and rotary  fixed and rotary wing  fixed and rotary wing  fixed and rotary wing  fixed and rotary wing  fixed and rotary wing | primary and secondary retrieval  primary and secondary retrievals  primary and secondary retrieval  primary and secondary retrieval  primary and secondary retrieval  primary and secondary retrieval  primary and secondary retrieval  primary and secondary retrieval  primary and secondary retrieval  primary and secondary retrieval  primary and secondary retrieval, organ transport  secondary retrieval, international transportation  primary and secondary retrieval  primary and secondary retrievals  primary and secondary retrievals  primary and secondary retrievals  primary and secondary retrievals  primary and secondary retrievals  primary and secondary retrievals  primary and secondary retrievals  primary and secondary retrievals  primary and secondary retrievals  paediatric and neonatal patient (newborns, to critically ill babies, kids and teens) retrievals  paediatric and neonatal patient retrievals  primary and secondary retrievals  primary and secondary retrievals of critically ill neonatal and paediatric patients  primary and secondary retrievals  primary and secondary retrievals of adult, neonatal and paediatric patients  primary and secondary retrieval of adults, paediatric, obstetric and neonatal critical care  primary and secondary retrieval of adults, paediatric, obstetric and neonatal critical care  primary and secondary retrieval of adults and neonates  primary and secondary retrieval of adults, neonates and paediatrics  primary and secondary retrieval of neonates, children, adolescents and expectant mothers  primary and secondary retrieval of adults and children  primary and secondary retrieval  primary and secondary retrievals of adult and paediatric patients  primary and secondary retrievals of adult and paediatric patients  primary and secondary retrieval  secondary retrievals only  primary and secondary retrievals for adults, children and newborn  primary and secondary retrievals  primary and secondary retrievals  primary and secondary retrievals of paediatric and neonatal patients  secondary retrievals only for neonatal,  paediatric patients  and adults  primary and secondary retrievals  primary and secondary retrievals  primary and secondary retrievals | registered nurse and paramedic  registered nurse and paramedic  registered nurse and paramedic  registered nurse, paramedic respiratory therapist  registered nurse and paramedic  registered nurse and paramedic  registered nurse and paramedic or two flight nurses  registered nurse  registered nurse and paramedic  registered nurse and paramedic  registered nurse and paramedic  registered nurse and respiratory therapist  registered nurse and paramedic  registered nurse, paramedic, respiratory therapist  paediatric emergency  registered nurse, paediatric respiratory therapist and paediatric emergency medical technician  registered nurse, paramedic, respiratory therapists, neonatal and maternal registered nurse  paediatric registered nurse, respiratory therapists and paramedics  registered nurse, paramedics, respiratory therapists  registered nurse, paramedics, respiratory therapists  registered nurse and paramedics  registered nurse and paramedics  registered nurse and paramedics  neonatal/paediatric registered nurse, respiratory therapists and paramedics  registered nurse and respiratory therapists with special training and experience in paediatric emergency care  registered nurse and paramedics  registered nurse and respiratory therapists with special training and experience in paediatric emergency care  registered nurse and paramedics  registered nurse, paramedics, respiratory therapists  registered nurse, paramedics, respiratory therapists  registered nurse and respiratory therapists  registered nurse and/or paramedics  registered nurse, neonatal and paediatric specialists, paramedics, and respiratory therapists  registered nurse, respiratory therapists, paramedics and emergency medical technicians  registered nurse, neonatal registered nurse, paramedics, and registered respiratory therapists.  registered nurse and paramedic  registered nurse and paramedic  registered nurse and paramedic  registered nurse and paramedic  registered nurse and respiratory therapist  registered nurse, paramedic, neonatal registered nurse, neonatal intensive care registered nurse, obstetrical registered nurse, and respiratory therapist  registered nurse and paramedic  registered nurse and paramedic  registered nurse and respiratory therapist  registered nurse paediatric registered nurse, respiratory therapist and paramedic  registered nurse and paramedic  registered nurse, paramedic, respiratory therapist  nurse and paramedic | Life Flight Network, a not-for-profit air medical transport service in the United States, is owned by the consortium of Oregon Health & Science University, Legacy Health, Saint Alphonsus Regional Medical Centre and Providence Health & Services. Life Flight Network offers ICU-level care during air transport across the Pacific Northwest and Intermountain West and Alaska. Shares a collaborative partnership with the Good Shepherd Health Care System, Samaritan Healthcare and Providence Health Care. Flight nurses are trained in RN, NRP, BSL, PHTLS, CEN, TPATC, ACLS, PALS, NRP plus 5 years ICU/ED experience). Paramedics are trained in BLS, NRP, FP-C, ACLS, PHTLS, PALS plus 5 years ICU/ED experience. Paramedics with certifications in BLS, NRP, FP-C, ACLS, PHTLS, PALS.  University of New Mexico Hospital’s Lifeguard Air Emergency Services provide adult, neonatal and paediatric emergency care And telemedicine services. Lifeguard operates throughout New Mexico, eastern Arizona, south-eastern Utah, southern Colorado and western Texas. Aircraft is owned by partner, SevenBar Aviation. Crews are specially trained nurses and paramedics.  https://unmhealth.org/services/emergency-trauma/lifeguard.html  Mercy Flights provides nonprofit air and ground medical transport in Southern Oregon and Northern California. The two-person, in-flight medical staff includes a specially trained registered nurse and paramedic equipped with advanced life-support systems.  Weblink: https://www.mercyflights.com/flight/  https://vfrg.casa.gov.au/emergency-procedures/mercy-flights/  https://www.mercyflight.org/mercy-flight-ems/  Atrium Health’s MedCenter Air provides emergency patient transport and support Atrium Health is an integrated, nonprofit health system in North Carolina, Georgia and Alabama. Registered nurse, respiratory therapists and paramedics trained in intensive care, emergency/critical care. Other requirements include TNCC, ITLS, ATLS, ACLS, PALS, BLS, STABLE. The fixed wing aircraft are operated by GAMA Aviation, LLC.  https://atriumhealth.org/medical-services/specialty-care/other-specialty-care-services/medcenter-air  North Flight Aero Med is a joint venture between two charitable health care organizations (Spectrum Health Hospitals and Munson Healthcare) operated by Spectrum Health Hospitals. Medical team members are certified in Advanced Cardiac Life Support (ACLS), pediatric (PALS), neonatal (NRP), and trauma care.  Weblink: https://northflightaeromed.org/ https://northflightaeromed.org/  Regional Emergency Medical Service Authority (REMSA) is a private nonprofit community-based service which is solely funded by user fees with no local community tax subsidy. Care Flight, a program of REMSA, is a private non-profit community-based service which is solely funded by user fees and no local community tax subsidy. Care Flight provides services throughout northwest Nevada and northeastern California region. Medical teams have qualifications in Certified Flight Nurse (CFRN) and the Critical Care Paramedic (CCP) certificate.  <https://www.remsahealth.com/about-us/>  AIRLIFE Denver is the Emergency Medical /Critical Care Transport Service of the HealthONE system of hospitals, clinics and healthcare plazas providing air and ground critical care transport for both adult and paediatric medical/ trauma patients.  https://airlifedenver.com/  Airlift Northwest Air Medical Service is a not for profit program of the University of Washington. Airlift Northwest's pilots, mechanics and aircraft are supplied, trained and maintained by outside contractors: Air Methods Corporation and Aero Air, LLC. Airlift Northwest has a partnership agreement with the Mary Bridge Children’s and Tacoma General hospitals, Jefferson Healthcare and the MultiCare Health System to transport paediatric and neonatal patients. Registered nurses are trained in critical care and emergency room care. Nurses can also have qualifications in Basic Life Support (BLS), Advanced Cardiac Life Support (ACLS), Paediatric Advanced Life Support (PALS). Neonatal Resuscitation Program (NRP), Advanced Trauma Care for Nurse (ATCN).  https://www.uwmedicine.org/airliftnw  Indiana University Health LifeLine is a not-for-profit hospital-based program that operates in Indiana and portions of Illinois, Kentucky, Michigan and Ohio. Staff are trained in Adult/Pediatric Critical Care, Neonatal/Paediatric Critical Care, Advanced Life Support and Basic Life Support Advanced Cardiac Life Support (ACLS), Paediatric Advance Life Support (PALS), Basic Life Support (BLS), Neonatal Resuscitation (NRP), Trauma certification (i.e. PHTLS, TNCC, TPATC, ATCN). Certifications include Advanced Flight Certification (CFRN for RNs and FP-C for Paramedics).  Weblink: <https://iuhealth.org/for-providers/lifeline>  Grace On Wings is a non-profit public charity organization under IRS code 501(c)(3) that provides charity air ambulance services within the United States and is based out of the Indianapolis International Airport.  https://www.graceonwings.org/  https://www.graceonwings.org/  The not for profit Methodist Health System based in Texas is affiliated by agreement with the North Texas Conference of the United Methodist Church. Methodist Health System is the founding member and sponsor of CareFlite air. CareFlite is a Texas, 501(c)3 nonprofit corporation sponsored by Baylor Scott and White, JPS Health Network, Methodist Health System, Parkland Health and Hospital and Texas Health Resources. CareFlite offers a membership program that protects families against balance billing after transports. The program covers individuals or households.  https://www.careflite.org/fixedwing.aspx  Critical Care Transport is a University of Alabama Hospital-based, inter-facility transport program for patients in the United States and worldwide who require transport from one medical facility to another. Each team member is trained in aeromedical physiology. For neonatal patients, a neonatal nurse practitioner or neonatal ICU RN and respiratory therapist will be part of the transport care team.  https://www.uabmedicine.org/patient-care/treatments/critical-care-transport  https://www.uabmedicine.org/patient-care/treatments/critical-care-transport  Air St. Luke’s covers Southern and Central Idaho, Eastern Oregon, and Northern Nevada from our bases in Boise and Twin Falls. Teams are trained to manage critical care patients across all age groups. Nurse with ICU/ED experience, Basic Life Support (BLS), ACLS, Paediatric Advanced Life Support (PALS), neonatal resuscitation (NRP), TPATC. Specialties include RN and respiratory therapist (RT), trained to manage critical care maternal and paediatric patients. Fixed wing services provided by Turbo Air, Inc. St Luke’s is a not-for-profit health system that relies on donations and memberships, volunteering  University of North Carolina Air Care provides prehospital and interfacility emergency care for adults, neonate and paediatric services. Fixed wing aircraft are operated by Jet Logistics Inc. The fixed wing aircraft provide repatriation from other states, and internationally such as Canada, Mexico, and the Caribbean.  https://www.uncmedicalcenter.org/uncmc/care-treatment/emergency-care/carolina-air-care/  The not-for-profit paediatric healthcare network provides emergency medical transport services for infants, kids and teens aged from birth to age 18 across Colorado. Staff are registered nurse specializing in neonatal medicine, paediatric medicine, and paediatric critical care. Children’s One emergency fixed wing transport is provided by Guardian Flight.  https://www.childrenscolorado.org/doctors-and-departments/departments/emergency-transport/  HELP Flight is a hospital-based air medical transport program offered by St. Vincent Healthcare of Billings, Montana. The St. Vincent Healthcare air medical program caters for adult and paediatric patient transportation. The fixed wing is operated by Edwards Jet Center. Nurse and paramedics are trained in critical care and emergency pre-hospital transport. Certifications include CFRN (Certified Flight Registered Nurse) or CCRN (Critical Care Registered Nurse).  https://www.sclhealth.org/locations/st-vincent-healthcare/services/emergency/help-flight/  Paediatric And Neonatal Doernbecher Transport (PANDA) provides care for critically ill and injured neonates and paediatric patients from newborn to age 18 during inter-facility fixed wing transportation. Doernbecher is one of Oregon’s children’s hospital in the United States and part of Oregon Health and Science University. Staff trained in emergency medical care and paediatric intensive care and/or neonatal intensive care. Other qualifications include Certified Flight Registered Nurse or Certified Neonatal Paediatric Transport. Fixed wing transport is contracted from Life Flight Network and AirLink.  The Neonatal and Paediatric Specialty Transport Services is run by Children's Health's. The transportation service covers neonatal, obstetric and paediatric patients also telehealth. Clinical team comprises a neonatal/paediatric team with certifications including BLS, ACLS, PALS and NRP, as well as advanced trauma certifications such as ATCN, ATLS or TNCC,.  https://www.childrens.com/  Sanford AirMed is a part of the non-profit Sanford Health’s integrated system of care. Sanford AirMed specialises in adult, neonatal, obstetric and paediatric care, trauma and organ transportation. Staff are certified in multiple disciplines of emergency medicine, advanced invasive procedures, and advanced ICU skills.  https://www.sanfordhealth.org/  Vanderbilt LifeFlight is part of Vanderbilt University Medical Centre and the Monroe Carell Jr. Children's Hospital. Vanderbilt LifeFlight's air operations are provided by Air Methods Corporation. All medical staffing, patient care and clinical services are provided by Vanderbilt University Medical Centre.  https://www.vumc.org/lifeflight  STAT MedEvac is a critical care transport system and is the clinical arm of the Centre for Emergency Medicine of Western Pennsylvania (CEM). CEM is a not-for-profit organization run by a consortium of hospitals; UPMC Presbyterian Shadyside, UPMC Children’s Hospital of Pittsburgh, UPMC Hamot, UPMC Altoona, and UPMC Mercy Hospital. Fixed wing aircraft is provided by Direct Air Carriers. Staff is provided by CEM. Paramedic qualifications include: Nationally Registered EMT-P, ACLS, ITLS, CPR, PALS, NRP. Nurse qualifications include: RN with extensive critical care unit or emergency department experience (3 year minimum), ACLS, ITLS, CPR, PALS, NRP. BTLS.  Avera Careflight is the medical arm of Avera, a health ministry of the Benedictine Sisters of Yankton and the Presentation Sisters of Aberdeen, both in South Dakota.  https://www.avera.org/services/emergency-trauma/careflight/  Cook Children's Transport Services is the emergency transportation arm of the not for profit Cook Children's Medical Centre. The transport service supports children with life-threatening illnesses or injuries. including treatment for illnesses such as multi-system trauma, respiratory distress/failure, infections and shock.  https://www.cookchildrens.org/services/transport/  Dell Children’s Medical Centre’s Paediatric Critical Care Transport Team partners with Air Medical Inc. to provide fixed-wing aircraft and pilot services for long distance air transport for paediatric patients in the Central Texas area of the US. Staff is trained in trained in advanced neonatal life support and has advanced airway skills.  https://www.dellchildrens.net/for-healthcare-professionals/neonatal-specialty-transport-services/  Owned by the not-for-profit Memorial Hermann Health System of Southeast Texas, Memorial Hermann Life Flight operates as a hospital-based, non-profit emergency transport service that relies on community support and fundraising efforts to pay for the service.  https://memorialhermann.org/about-us/our-organization  Critical Care Transport Program of Boston Children's Hospital provides resuscitation, stabilization, and inter-hospital transfer of patients ranging from preterm infants to young adults. Aircraft is provided by private vendor, Boston MedFlight,  https://www.childrenshospital.org/about-us  https://www.childrenshospital.org/centers-and-services/programs/a-_-e/critical-care-transport-program  Boston MedFlight is a nonprofit organization that provides critical care medical transport by air and ground for critically ill and injured infants, children and adults. Boston MedFlight is operated by a consortium of Boston's seven leading hospitals. Aircraft is operated and maintained by SevenBar Aviation.  https://www.bostonmedflight.org/medical-center-consortium/  https://www.bostonmedflight.org/medical-airplane-transport/  Based at the University of Utah Hospital, AirMed provides air transport for patients from Colorado, Wyoming, Nevada, Montana, Idaho and the state of Utah. AirMed flight nurse have a minimum of five years’ experience in an ICU or ED. paramedics have a minimum five years’ experience with a high-volume 911 agency. Respiratory therapists have a minimum of two years’ experience in an ICU and ED at University of Utah Health. All flight positions must have additional certifications that include, but are not limited to, ACLS, PALS, NRP, and BLS.  https://healthcare.utah.edu/airmed/  Intermountain Healthcare is a Utah-based, not-for-profit system of 24 hospitals. Intermountain Life Flight is a nonprofit healthcare organization meaning pilots, crew, and support staff are all employed by Intermountain Healthcare. Aircraft are also owned and operated by Intermountain Healthcare  https://intermountainhealthcare.org/accessing-care/emergency-services/life-flight  SURVIVAL FLIGHT, the critical care transport program of Department of Emergency Medicine of the Michigan University, provides rapid and safe transport of critically ill and injured patients of any age group. Flight nurses are dually licensed as paramedics. Aircraft are provided by Metro Aviation who also supply flight crew and is responsible for control of all flight operations.  http://www.med.umich.edu/survival_flight//about/index.html  Life Link III is a not for profit consortium comprising ten healthcare organizations including the University of Minnesota. Clinical crew have experience in hospital intensive care, emergency room or 911 experience and certified flight registered nurse and paramedics. Life Link III hosts LinkED, a regional education series which focuses on providing continuing clinical and safety education (CE) to industry partners.  https://www.lifelinkiii.com/aircraft/fixed-wing/  Mayo Clinic is a not for profit organisation that relies on generous donations and grants. Each flight is staffed with at least two medical professionals who have specific training in flight safety and flight physiology and who have multiple education certifications, including Advanced Cardiovascular Life Support.  https://www.mayoclinic.org/ambulance-service/air-ambulance/airplane  Clinical staff are advanced life support trained. The provision of fixed wing aircraft is outsourced from PHI Health, a FAA Part 135 certificate holder.  https://www.childrensmercy.org/health-care-providers/transport/  The hospital-based Benefis Mercy Flight operates in regions including Spokane and Seattle, Washington; Salt Lake City, Utah; and Denver, Colorado. Fixed wing air transport are contracted through Aero Air. Benefis Health System is a not for profit consortium of Benefis Sletten Cancer Institute, Heart and Vascular Institute, Orthopaedic Centre of Montana, women’s & children’s services and emergency medicine in Montana.  https://www.benefis.org/services-specialties/emergency-services/emergency-services  https://www.benefis.org/services-specialties/emergency-services/mercy-flight  Air Link is an air ambulance service of the community-based Regional West Health Services in West Nebraska. The air ambulance services the region including Nebraska, Wyoming, South Dakota, Kansas, and Colorado.  https://www.rwhs.org/services/trauma-emergency/air-link  A partnership program of Ascension Borgess Hospital and Bronson Methodist Hospital Staff are registered nurse with ICU/ED experience and are also licensed paramedics. Aircraft is provided by Metro Aviation Inc which maintains exclusive operational control over all aircraft whilst West Michigan AirCare provide the medical personnel. Staff are qualified in Advanced Cardiac Life Support (ACLS), Paediatric Advanced Life Support (PALS), Neonatal Resuscitation (NRP) and Prehospital Trauma Life Support (PHTLS). Nurse are also required to complete a Certified Flight Registered Nurse (CFRN) examination; as well as the Flight Nurse Advanced Trauma Course.  http://www.aircare.org/about/medical-crew/  Beacon Health System is a not-for-profit community-governed health service organisation incorporating multiple hospitals in Indiana including Beacon Children’s Hospital, Beacon Granger Hospital, Community Hospital of Bremen, Elkhart General Hospital, Epworth Hospital, Three Rivers Hospital and Memorial Hospital of South Bend. Staff are certified holders of ACLS, PALS, STABLE, TNCC, BTLS/PHTLS, FP-C or CFRN. https://www.airmethods.com/about-us/divisions/ Advanced Cardiac Life Support, Paediatric Advanced Life Support  San Juan Regional Medical Centre is a non-profit hospital serving the Four Corners area of New Mexico, Arizona, Colorado, and Utah. Fixed wing aircraft is hospital owned and operated by Air Methods.  https://www.sanjuanregional.com/ourservices/aircare  Angel One Patient Transport program is owned and operated by Arkansas Children's Hospital. The program provides transport to patients up to 21 years of age. Staff area trained in Advanced Cardiac Life Support (ACLS), Paediatric Advanced Life Support, Neonatal Resuscitation Program and nationally certified in neonatal-paediatric transport (C-NPT) including Paediatric Intensive Care (PICU) and Neonatal Intensive Care (NICU).  https://www.archildrens.org/  CareFlight of the Rockies is an air transport service of St. Mary’s Medical Centre that operates in Western Colorado and Eastern Utah. Registered nurses are trained and certified in advanced cardiac life support (ACLS), paediatric advanced life support (PALS) or the emergency nurse paediatric course (ENPC.)  https://www.sclhealth.org/locations/st-marys-medical-center/services/emergency/  Flight For Life® Colorado is an emergency air transport program of Centura, a not for profit, Christian-based partnership organisation of not for profits CommonSpirit Health (Catholic) and AdventHealth (Seventh-day Adventist) health care organizations in the southern and southwestern regions of the United States, All Flight For Life® Colorado fixed wing aircraft are operated by Mayo Aviation, Inc. Staff are trained in ALS, PALS NRP (nurse) and ALS (paramedics).  https://www.centura.org/our-ecosystem/flight-for-life/about-us  Cleveland Clinic's Critical Care Transport (CCT) is operated by not for profit group practice, Cleveland Clinic. Caters for neonate and adult retrievals. Fixed wing aircraft is provided by private company, Aery Aviation LLC.  https://my.clevelandclinic.org/departments/critical-care  Nationwide Children’s Hospital's Critical Care Transport is a hospital based air transport program of the Nationwide Children’s Hospital, a not for profit paediatric hospital in Ohio.  https://www.nationwidechildrens.org/specialties/transport  https://www.nationwidechildrens.org/about-us/our-story/hospital-overview  The LifeFlight® critical care transport service is a program of Nicklaus Children’s Hospital based in Miami, Florida and also covers South Florida, the Caribbean, and Central and South America. Fixed-wing jets are contracted on demand for long-distance transports.  https://www.nicklauschildrens.org/  Metro Life Flight is a not-for-profit, CAMTS-accredited, air and ground critical care transport service. Metro Life Flight’s aircraft are operated by Metro Aviation Incorporated. MetroHealth is an academic medical center that operates four hospitals, four emergency departments and more than 20 health centers and 40 additional sites throughout Cuyahoga County.  SCL Health is a faith-based, nonprofit healthcare organization that operates across Colorado, Montana, and Kansas.  Air Link is an air transport service of Regional West tertiary referral medical center that provides health care services across western Nebraska and the neighboring states of Colorado, South Dakota, Kansas and Wyoming. RWHS is a non-profit corporation that is organized as a parent company for affiliated non-profit health care organizations. |
| United Kingdom (UK) | Scottish Air Ambulance  Scottish Specialist Transport and Retrieval (ScotSTAR)  The Isle of Man Department of Health and Social Care  Channel Islands of Guernsey and Jersey Air Ambulance  Embrace Yorkshire & Humber Infant & Children's Transport Service | government funded  not for profit (donations, government grants, commercial insurance, medical insurance, private payers, corporate sponsors, fundraising)  government funded  government funded  government funded  government funded | fixed and rotary wing  fixed and rotary wing  fixed-wing only  fixed-wing only  fixed and rotary wing | primary and secondary retrieval  primary and secondary retrieval  secondary retrieval  secondary retrieval  primary and secondary retrievals of neonatal and paediatric patients | paramedic  doctor, registered nurse and paramedic  paramedic  paramedic  doctor, registered nurse and neonatal nurse | The Scottish Ambulance Service is part of NHS Scotland. The service has the only government-funded air ambulance service in the UK operated under contract by Gama Aviation.  https://www.scottishambulance.com/our-services/emergency-care/  ScotSTAR was established in April 2014 as the dedicated paediatric intensive care transport with the Scottish Ambulance Service, Paediatric Retrieval Service and Neonatal Transport Service. The ScotSTAR incorporates paediatric and neo-natal retrieval and emergency retrieval services and is based at Glasgow Airport. The Scottish Neonatal Transport Service is part of ScotSTAR and is a specialist service dedicated to the safe transport of unwell newborn infants throughout Scotland and on occassion when Scottish babies require transferred further afield.  Weblink: https://www.neonataltransport.scot.nhs.uk/https://www.scottishambulance.com/our-services/neonatal-care/  The Isle of Man Department of Health and Social Care operates a fixed-wing air ambulance for patient transport between the island and mainland hospitals  https://www.gov.im/about-the-government/departments/health-and-social-care/  The Channel Islands of Guernsey and Jersey have a Beechcraft King Air B200 air ambulance operated by Gama Aviation (AIM:GMAA).  https://www.gov.je/news/2020/pages/AirAmbulanceGama.aspx  Part of Sheffield Children's NHS Foundation Trust, is a neonatal and paediatric transport service in the UK operating within the Yorkshire & Humber regions. . All Embrace staff have completed NLS and APLS courses and the majority have  attended a transport course, eg PaNSTaR (Paediatric and Neonatal Safe Transfer and Retrieval course).  http://www.embrace.sch.nhs.uk |
